# Teaching Outside the Lines: Using Art to Enhance Mental Status Exam Skills

**DOI:** 10.1007/s40596-025-02258-9

**Published:** 2025-11-04

**Authors:** Jennifer Siegel, Kai-Hong Jeremy Mao

**Affiliations:** Keck School of Medicine of USC, Los Angeles, CA US

Integrating arts and humanities into graduate medical education is an active area of research [1, 2]. The arts provide medical students with improved clinical observation skills, diagnostic reasoning, and empathy [1, 3–5], which are critical for effective and impactful patient care. In fact, the Association of American Medical Colleges recently stated the need for arts and humanities in medical education, along with strategies to incorporate them [2].

Visual thinking strategy, a structured discussion-based method of art interpretation (not art-making-based), is one type of arts-based curricula actively being researched, with noted benefits in medical education; however, the majority of these courses are elective in nature (likely attracting students already interested in medical humanities) [6]. More generally, no arts-based curricula to our knowledge specifically target medical students actively participating in clerkships, where the application of such clinical skills may be especially tangible [7].

The field of psychiatry may be a particularly amenable area to further develop the application of arts-based clinical educational materials. A recent systematic review examining humanities programs within psychiatric education identified 35 articles considering potential uses of arts in this field, consistent with broader calls for integration of humanities into medical education. Most of these applications focused on film and media, rather than visual arts, leaving further room for varying applications of humanities into psychiatric education [2]. Studies exploring innovations in psychiatric education have shown positive reception by students, increased potential for learning and engagement, and improvements in their attitudes towards psychiatry [8]. Other studies have shown that discussions surrounding unconscious bias towards those with mental illness can be facilitated through observing artworks made by or depicting those with mental illness [9–12]. Further, more researchers are acknowledging the need to integrate the arts specifically into psychiatric education and emphasizing the justification for such research [2].

The mental status exam (MSE) is the key diagnostic tool in psychiatric assessments, where descriptions and observations of patients are of critical importance. Despite there being research calling for innovations in psychiatric education as a means to learn new material [2, 8], there is sparse literature regarding innovative means for teaching and practicing the MSE [13, 14], making novel curricula related to the MSE of particular interest. Given that students tend to stigmatize mental illness more than psychiatrists, but less than other physicians [12], and that their psychiatry clerkship may be their only significant exposure to psychiatry in a clinical setting, this type of education has the added potential benefit to shape future physicians’ attitudes towards mental health.

Our educational innovation examined how the arts may enhance descriptive and observational skills, as related to the MSE. Our goal was to pilot a high-yield, time-efficient artistic didactic experience with an art-making component that can be easily incorporated as an educational tool for students rotating through their core psychiatry clerkship. Additional goals included helping students improve their sense of descriptive and observational skills, recognizing the benefits of the humanities in medical education, enjoying their experience, and shaping potential attitudes surrounding mental health.

## Project Design

All third-year medical students at the Keck School of Medicine of USC were asked to participate in a 1-h arts-based module, scheduled at the beginning of their core psychiatry clerkship. This workshop was held during their weekly required didactic time on Zoom. Over the course of one academic year, there were eight cohorts of rotating medical students, with each cohort containing roughly 24 students. Thus, this workshop was provided eight times throughout the year. The instructor was the Chief Resident in Psychiatry, who has a background as an artist and led similar modules at academic institutions. Workshop participation had no influence on grades. All assessments and feedback were obtained anonymously. Students were asked to create a unique anonymous code (i.e., birth date and favorite color—March 12th, green = 0312green) so that pre- and post-lecture data could be linked by individual. Data collected online remained password protected. An IRB was submitted and exempt under category 1. All students received an information sheet and recruitment sheet for this voluntary study before the lecture.

The 1-h module consisted of two exercises. The first exercise, “Drawers & Describers,” focused on teaching description skills (see Table 1). Students were paired off into virtual breakout rooms with one being the “drawer” and the other the “describer,” the latter of whom described a landscape image as the other utilized descriptors to sketch a corresponding picture. Roles switched after 10 min using a different painting. Once completed, the students came back together and the source images were revealed, leading to a thorough discussion about what made the exercise easy or challenging. Students also shared their drawings for visual comparisons regarding similarities and/or differences between each image interpretation. Students then applied concepts discussed to examples from their clinical encounters, highlighting the importance of descriptive accuracy.

**Table 1:** Arts-based activity information

Activity	Recommended materials	Curricular goal	Activity duration	Activity adapted from
“Drawers & Describers”	Two different landscape paintings (i.e., *A Coming Storm by Sanford Glifford* (1863) and *Pichincha by Frederic Edwin Church *(1867)), a sheet of paper, and a drawing instrument (i.e., pen, pencil, colored pencils, etc.). Can be modified to be drawn on an electronic device. Landscape images were selected, rather than human figures, as they provide a more accessible subject matter for practicing descriptive skills via drawing	Descriptive Skills	~ 30 min	A preclinical elective at UT Southwestern’s Medical School [15]
“Looking for 30 Seconds”	A painting with a single human figure (i.e., *The Annunciation* by Henry Ossawa Tanner (1898)), pencil, and a sheet of paper. Can alternatively take notes on an electronic device	Observation skills	~ 20 min	Harvard Graduate School of Education’s Project Zero [16]

Exercise 2, “Looking for 30 Seconds,” focused on observational skills. Everyone observed a painting for 30 s, first as a whole and then by quadrants (looking at each quadrant for 30 s, for a total of 2 min), writing down their observations. Students were asked to use that information to infer what an MSE for the woman in the painting might look like (i.e., soft speech due to reserved body posture). This led to a discussion focused on observation skills, as related to formulating an MSE, and again was applied to real clinical encounters.

Prior to this 1-h module, students were asked to complete a seven-item electronic survey (~ 3 min), ranking their level of agreement on a 5-point Likert scale from 1 (“strongly disagree”) to 5 (“strongly agree”) with certain statements. The questions were generated for this pilot and assessed students’ attitudes relating to self-descriptive skills, self-observation skills, and perceived benefits of integrating humanities into psychiatric education. After class, students repeated the same survey but with six additional Likert scale questions and two soliciting open-ended positive and negative feedback of the session (~ 5 min). Paired *t*-tests (*p* value < 0.05) examining the significance of the improved scores post-intervention, as compared to pre-intervention, analyzed repeated survey questions, while open-ended feedback was acknowledged for future course iterations.

### Learning Gains and Reflections

In total, 163/190 (86%) of students completed the pre-workshop survey, and 127/190 (67%) of students completed the post-workshop survey. Compared to averaged pre-workshop agreement scores for each statement, all averaged post-workshop scores for all statements showed an increase in strength of agreement. Of those that completed the pre- and post-workshop surveys, 113 students completed both surveys, identified by anonymous codes. Paired analysis revealed statistically significant differences in answers to all statements in an improved direction. Most notably, the following were found: “I am comfortable completing an MSE independently” (3.43 vs 3.83, *p* < 0.0000001); “I am comfortable observing psychiatric patients’ behaviors” (3.86 vs 4.07, *p* < 0.05); “I am comfortable describing psychiatric patients’ behaviors” (3.38 vs 3.82, *p* < 0.00000001); “I am detailed when I write a MSE” (3.62 vs 3.87, *p* < 0.00004); “I am detailed when I present a MSE” (3.48 vs 3.86, *p* < 0.00000007); “I am able to utilize my observation skills to construct a complete and descriptive MSE” (3.52 vs 4.0, *p* < 0.000000001); and “arts skills training is beneficial to medical education” (3.68 vs 4.02, *p* < 0.003).

Average scores on the 5-point Likert scale from 1 (1 = strongly disagree) to 5 (5 = strongly agree) for the 127 students that completed the post-workshop survey demonstrated overall enjoyment of activities (4.22), finding the activities educational (4.0), feeling like the activities helped improve MSE skills (3.65), seeing value in the “Drawing & Describing” exercise (3.9) and the “Looking for 30 Seconds” exercise (3.76), and reporting that they would recommend this module to future psychiatry students (3.90).

The group discussions for the “Drawers & Describers” exercise highlighted different approaches to convey the scene, with some strategies setting the overall scene first, while others focused on specific details (see Table 2). Students noted the weight of initial descriptions anchoring them to an imagined scene. In comparing student drawings, students noted that often prominent features were conveyed in each interpretation, but how those prominent details were conveyed varied. In applying these lessons clinically, discussion focused on how variations in describing patients in their MSE (i.e., on rounds, in written notes) could influence subsequent case formulations, treatment decisions, and patient outcomes.

**Table 2:**
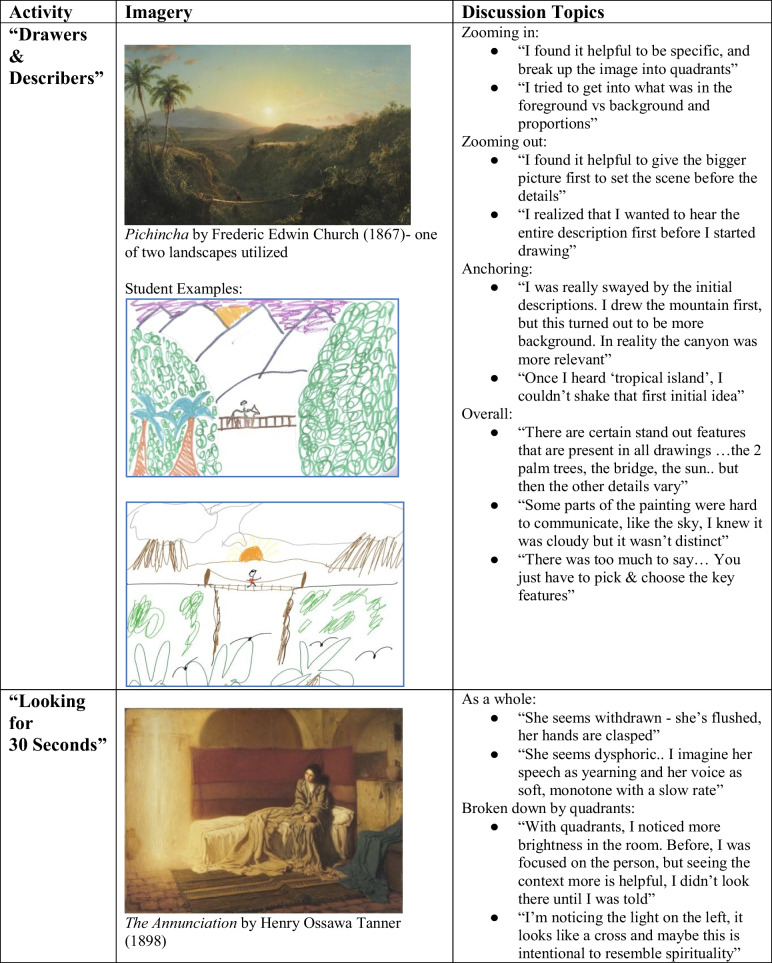
Arts-based images and discussion themes

Both images of famous artwork included in this manuscript (The Annunciation by Henry Ossawa Tanner (1898) and Pichincha by Frederic Edwin Church (1867) are part of the public domain (due to their age) and widely available online. They have no copyrights associated with these works as they are part of the public domain. There are 2 student artwork examples included. All students were informed in the consent process by both study information sheet and recruitment email that if they volunteered to share their artwork created during the psychiatry lecture, it could be used anonymously in any potential future publication or presentation. Both images were then voluntarily emailed to the lecturer after the session

The group discussions for the “Looking for 30 Seconds” exercise highlighted how observations may vary when one is evaluating the big picture versus specific details. Students provided clinical examples of how noting overall context and observing particular components of that context have come together to not only inform diagnostic and treatment decisions, but also allow one to build rapport with patients, which may then further enhance psychiatric assessments and the implementation of those treatments.

In general, students provided positive feedback, describing the workshops as “fun,” “unique,” “motivating,” and “helpful.” Some students noted wanting more integration of the arts into medical education, while others emphasized better conceptualizing the “importance of communicating,” with one student writing, “Thank you for reminding me of the value of a descriptive, detailed MSE. I think it’s tempting to fall into a habit of using general descriptors in my write ups and presentations, but I’m motivated by your workshop to break that habit.”

Constructive feedback focused on the timing of the workshop, in terms of requesting more than 1 h for the session, holding it on a day with fewer lectures, and having it be one of the first lectures of the clerkship. Others expressed wanting to apply these exercises to mock or real patient encounters.

## Looking Ahead

Students’ improved sense of both their descriptive skills and observation skills after the workshop, as compared to before the workshop, was statistically significant. Students were able to more tangibly grasp the importance of descriptions via the application of concepts through drawing and see visually how descriptions influence interpretations. Similarly, students were able to consider the potential impact of their observation skills on clinical care concretely via discussion. The whole scene allows one to incorporate a patient’s environment into diagnostic reasoning. This can be expanded beyond the face-to-face encounter, where context more globally (i.e., more systemic and societal factors) also contributes to the clinical picture. For example, is a patient truly “non-compliant” with medication, or are they unable to fill their prescription due to access issues (i.e., limited time off work during pharmacy open hours, lack of transportation)? More targeted observations also allow for one to notice alternate explanations for a patient presentation (i.e., Is the patient manic? And/or is the Red Bull sticking out of her backpack contributing?), alter subsequent care (i.e., focus on medication management vs counseling on energy drink side effects), or even help to build rapport (i.e., “I noticed your Dodgers tattoo, they’ve been playing well this season”), which may lead to more trust or openness.

The students’ opinions about the benefit of art in medical education significantly changed in favor of the arts after the workshop, as compared to before the workshop. In general, the workshop was overall received positively, with the majority of students finding the workshop educational, recommending it to future clerkship cohorts, and enjoying the workshop. The most prevailing constructive feedback was a desire to expand and/or refine the workshops. No participant suggested removing content. Additionally, it is important to note that this pilot was overall well received in a population (i.e., an entire class year of medical students) that did not actively seek a humanities experience and with the majority unlikely to pursue psychiatry as a future specialty. Thus, this type of programming may enhance engagement with psychiatric material despite one’s career path and suggests that it would be worthwhile to further develop.

It is important to note the limitations of this study. Not everyone considers themselves an artist. While it was emphasized that “artistic talent” was not the goal, and that if one was not able to fully draw an image from how it was described, they should focus on how they imagined or wanted to draw the image as a point of reference, it was not possible to eliminate variability in drawings based on drawing skill. Other limitations included that the focus of our study reflected changes in attitudes towards one’s descriptive and observational abilities, rather than known translation to actual improvements in descriptive and observational skills. In part, this was due to time constraints; however, we are hoping that this positive data serves to establish value in this type of work and lay the groundwork for future iterations with a control group and/or focused on the clinical translation of skills.

In summary, this pilot was the first known arts-based medical curricula designed for clerkship psychiatry students. This study was designed not only to develop a novel approach to teach fundamental content in psychiatric education, but to also further explore the benefits of incorporating the humanities into medical education more generally. Student feedback can be used to guide future iterations. In particular, we hope to implement a pre- and post-workshop mental status exam exercise assessing whether medical students are able to demonstrate improvements in the accuracy and comparability of their MSEs after completing the session. We hope that this pilot will serve as a guide for other institutions to design their own educational experiences for psychiatry clerkships. In addition, similar experiences could be envisioned to teach important clinical skills in non-psychiatry rotations, especially those that rely on detail-oriented visual skills, such as reading radiology scans or interpreting pathology slides. Finally, this experience complements the arts-based curricula that already exist for both pre- and post-clerkship students.

## Data Availability

The data that support the findings of this study are available from the corresponding author upon reasonable request.

## References

[1] Bell LT, Evans DJ. Art, anatomy, and medicine: is there a place for art in medical education? Anat Sci Educ. 2014;7(5):370–8. 10.1002/ase.1435. 24421251 10.1002/ase.1435

[2] Yaden ME, Sawaya RT, Reddy J, Jong KA, White J, Moniz T, et al. A systematic review of the arts and humanities in psychiatry education. Int Rev Psychiatry. 2023;35(7–8):540–50. 10.1080/09540261.2023.227818. 38461397 10.1080/09540261.2023.2278718

[3] Braverman IM. To see or not to see: how visual training can improve observational skills. Clin Dermatol. 2011;29(3):343–7. 10.1016/j.clindermatol.2010.08.001. 21496744 10.1016/j.clindermatol.2010.08.001

[4] Jasani SK, Saks NS. Utilizing visual art to enhance the clinical observation skills of medical students. Med Teach. 2013. 10.3109/0142159x.2013.770131. 23641917 10.3109/0142159X.2013.770131

[5] Gurwin J, Revere KE, Niepold S, Bassett B, Mitchell R, Davidson S, et al. A randomized controlled study of art observation training to improve medical student ophthalmology skills. Ophthalmology. 2018;125(1):8–14. 10.1016/j.ophtha.2017.06.031. 28781219 10.1016/j.ophtha.2017.06.031

[6] Cerqueira AR, Alves AS, Monteiro-Soares M, Hailey D, Loureiro D, Baptista S. Visual thinking strategies in medical education: a systematic review. BMC Med Educ. 2023;23(1):536. 10.1186/s12909-023-04470-3. 37501147 10.1186/s12909-023-04470-3 PMC10375761

[7] Mukunda N, Moghbeli N, Rizzo A, Niepold S, Bassett B, DeLisser HM. Visual art instruction in medical education: a narrative review. Med Educ Online. 2019;24(1):1558657. 10.1080/10872981.2018.1558657. 30810510 10.1080/10872981.2018.1558657 PMC6394328

[8] Sandrone S, Berthaud JV, Carlson C, Cios J, Dixit N, Farheen A, et al. Active learning in psychiatry education: current practices and future perspectives. Front Psychiatry. 2020. 10.3389/fpsyt.2020.00211. 32390876 10.3389/fpsyt.2020.00211 PMC7190786

[9] Cutler JL, Harding KJ, Hutner LA, Cortland C, Graham MJ. Reducing medical students’ stigmatization of people with chronic mental illness: a field intervention at the “Living Museum” state hospital art studio. Acad Psychiatry. 2012;36(3):191–6. 10.1176/appi.ap.10050081. 22751820 10.1176/appi.ap.10050081

[10] Economou M, Kontoangelos K, Peppou LE, Arvaniti A, Samakouri M, Douzenis A, et al. Medical students’ attitudes to mental illnesses and to psychiatry before and after the psychiatric clerkship: training in a specialty and a general hospital. Psychiatr Res. 2017;258:108–15. 10.1016/j.psychres.2017.10.009. 10.1016/j.psychres.2017.10.009 28992547

[11] Chavda P, Desai N. Attitudes of undergraduate medical students toward mental illnesses and psychiatry. J Educ Health Promot. 2018;7(1):50. 10.4103/jehp.jehp_87_17. 29693031 10.4103/jehp.jehp_87_17 PMC5903151

[12] Oliveira AM, Machado D, Fonseca JB, Palha F, Moreira PS, Sousa N, et al. Stigmatizing attitudes toward patients with psychiatric disorders among medical students and professionals. Front Psychiatry. 2020. 10.3389/fpsyt.2020.00326. 32425827 10.3389/fpsyt.2020.00326 PMC7207477

[13] Huline-Dickens S, Heffernan E, Bradley P, Coombes L. Teaching and learning the mental state exam in an integrated medical school. Part I: student perceptions. Psychiatr Bull. 2014;38(5):236–42. 10.1192/pb.bp.113.042655. 10.1192/pb.bp.113.042655 PMC4180989 25285223

[14] Martin A, Krause R, Jacobs A, Chilton J, Amsalem D. The mental status exam through video clips of simulated psychiatric patients: an online educational resource. Acad Psychiatry. 2019;44(2):179–83. 10.1007/s40596-019-01140-9. 31858445 10.1007/s40596-019-01140-9 PMC7082206

[15] Pitman, B, Goff H, Copeland A. The art of examination course spring 2019 UT. Southwestern Medical School. 2019. Accessed August 23, 2020, from https://www.utdallas.edu/arthistory/medicine/course/2019%20Art%20Exam%20Syllabus.pdf.

[16] Looking: ten times two. (2015). Project Zero. Harvard Graduate School of Education. Accessed August 23, 2020 from https://pz.harvard.edu/resources/looking-ten-times-two.

